# The Role of *Bifidobacterium bifidum* novaBBF7*, Bifidobacterium longum* novaBLG2 and *Lactobacillus paracasei* TJB8 to Improve Mechanisms Linked to Neuronal Cells Protection against Oxidative Condition in a Gut-Brain Axis Model

**DOI:** 10.3390/ijms241512281

**Published:** 2023-07-31

**Authors:** Sara Ferrari, Rebecca Galla, Simone Mulè, Giorgia Rosso, Arianna Brovero, Valentina Macchi, Sara Ruga, Francesca Uberti

**Affiliations:** 1 Laboratory of Physiology, Department of Translational Medicine, University of Piemonte Orientale, Via Solaroli 17, 28100 Novara, Italy; 2 Noivita Srls, Spin Off, University of Piemonte Orientale, Via Solaroli 17, 28100 Novara, Italy

**Keywords:** cognitive decline, gut-brain axis, probiotic, oral supplementation, gut microbiome

## Abstract

Despite the identification of several innovative targets for avoiding cognitive decline, there has yet to be a widely accepted approach that deals with minimising the deterioration of cognitive function. In this light, recent studies suggest that regulating the gut-brain axis with probiotics is a potential therapeutic strategy to support brain health. For this reason, in vitro models were used to examine the efficacy of different probiotic combinations to enhance intestinal homeostasis and positively affect the brain. Therefore, the new formulation has been evaluated for its capacity to modify intestinal barrier functions in a 3D in vitro model without any adverse effects and directly impact the mechanisms underlying cognitive function in a gut-brain axis model. According to our findings, *B. bifidum* novaBBF7 10 mg/mL, *B. longum* novaBLG2 5 mg/mL and *L. paracasei* TJB8 10 mg/mL may successfully modify the intestinal barrier and improve SCFA production. Successively, the probiotics studied caused no harm at the neuronal level, as demonstrated by iNOS, mitochondrial potential, and cell viability tests, confirming their safety features and enhancing antioxidant mechanisms and antineuroinflammation activity. Additionally, the damage caused by oxidative stress was also healed, and critical pathways that result in cognitive impairment were changed by synergetic action, supporting the hypothesis that brain ageing and neurodegeneration are slowed down. All these findings demonstrate the ability of probiotics to affect cognitive processes and their ability to sustain the mechanisms underlying cognitive function by acting on intestinal function.

## 1. Introduction

Cognitive functions are the most complex capabilities of the nervous system because they are responsible for rational perception, cognition, and interaction with the external environment; they are important in implementing some complex, knowledgeable tasks and the most routine household activities [1]. Cognitive impairment is particularly prevalent in the elderly age: according to statistics, dementia, a severe form of cognitive impairment, affects 3 to 20% of adults over the age of 65. Moreover, the prevalence of mild cognitive impairment among the elderly is much higher and ranges from 40 to 80%, depending on age. Neurocognitive impairment is an issue that concerns neurologists and other medical professionals because of the current trend towards longer life expectancies and, consequently, an increase in the population’s elderly [1]. From a clinical perspective, age-related cognitive decline is synonymous with changes in cognition and memory typical of growing older. Mild cognitive impairment (MCI) generally refers to a decline in learning new information or recalling stored information. However, six main cognitive domains may be affected (learning and memory, social functioning, language, visuospatial function, complex attention, or executive functioning) [2]. Regarding the risk factors, age is the main one for MCI. However, other risk variables include male sex, the presence of the apolipoprotein E allele, a family history of cognitive impairment, and several vascular risk factors, including hypertension, hyperlipidemia, coronary artery disease, and stroke [3]. Moreover, according to some research on multimorbidity and MCI, people who develop four or more chronic conditions, mainly two between hypertension, hyperlipidemia, coronary artery disease, and osteoarthritis, have the highest risk of developing this illness [4].

Additionally, lifestyle is important since it has been shown that cognitively and physically sedentary people are more likely to develop MCI [5]. At a molecular level, neuroinflammation, oxidative stress, and excitotoxicity are associated with several neurological disorders. In particular, different research suggests that accumulated oxidative stress may be one of the key mechanisms that cause cognitive impairment and neurodegenerative diseases, such as Alzheimer’s disease (AD) [6]. In addition, neuroinflammation can cause disruptions in synaptic transmission and glial and neuronal dysfunction that contribute to cognitive impairment; these changes include alterations in glutamate release, uptake, and clearance, as well as changes in the functions and subunit composition of its receptors [7].

Cognitive decline is a growing public health concern that refers to deterioration in mental ability to varying degrees and is very common in a multiplicity of conditions, including ageing, adiposity depression, and especially AD [8]. The major problem is that conventional treatments are limited and, nowadays, there are no drugs that can completely reverse symptoms: traditional therapeutic approaches can result, at best, in slowing the deterioration of cognitive function, but only if treatment is started at an early stage of the disease [9]. Considering this background, it is evident that finding treatments that promote and protect cognitive function is urgent. In this context, the gut microbiota has emerged in recent decades as a critical factor affecting neurophysiological and psychophysiological processes, including cognition, emotion neurotransmission, and neurodevelopment [10]. Specifically, the interaction between gut microbes and the central nervous system (CNS) exists within the gut-brain axis, a complex bidirectional association network between intrinsic gut microbes and the brain [11]. In detail, the gut microbiota indirectly and in a bidirectional way communicates with the brain through several pathways, including vagal nerve stimulation, interaction with the immune system, and microbial production of human neurotransmitters [12]. Psychiatric and concomitant nonpsychiatric illnesses are linked to dysregulation of gut-brain axis communication; a disruption of the molecular communication between the gut and the brain may result from these illnesses’ frequent associations with changes in the composition or function of the gut microbiota [13]. For instance, a rising number of studies in recent years have demonstrated that people with severe depressive illness have, a different gut microbiota composition compared to healthy controls [14]. A novel strategy to repristinate the gastrointestinal flora is the administration of probiotics, which are good living and transient entities that affect different pathways. Consuming probiotics has been demonstrated to enhance the expression of brain-derived neurotrophic factor (BDNF), a growth factor essential for memory, brain plasticity, and neuronal health that is severely low in depressed people [15,16,17]. Specifically, probiotics promote gut eubiosis and prevent cognitive function decline by decreasing amyloid-Beta (Aβ) levels in the hippocampus, reducing neuroinflammation, and maintaining neuronal structural integrity and plasticity [18]. Furthermore, oral administration of *Lactobacillus helveticus*, *Bifidobacterium longum*, and *Bifidobacterium breve* in a rodent model positively affected anxiety-like behaviours and strengthened memory [19]. Indeed, the administration of probiotics to rodents affected the gamma-aminobutyric acid (GABA) receptor and reduced stress-induced anxiety and depression-like behaviours [20]. Indeed, a combination of *L. helveticus* and *B. longum* has been shown to decrease or minimise the effects of anxiety and depression [21]. At the same time, another study reported how treatment with *Lactobacillus plantarum* showed anti-Alzheimer’s properties against D-Galactose-induced AD [22]. There is also evidence for the effectiveness of *Lactobacillus paracasei* in improving memory function through nerve growth factor-mediated neurogenesis [23]. Based on previous findings, it explores the effects of several probiotics to ameliorate cognitive activities. This study aimed to demonstrate this assumption by testing several probiotics in a gut in vitro model that mimics oral intake to reduce cognitive impairment. Furthermore, the key mechanisms underlying cognitive decline are explored in a model of induced neurodegeneration.

## 2. Results

### 2.1. The Effects of B. bifidum novaBBF7, B. longum novaBLG2 and L. paracasei TJB8 Supplementation on the In Vitro Barrier Model

**Figure 1:**
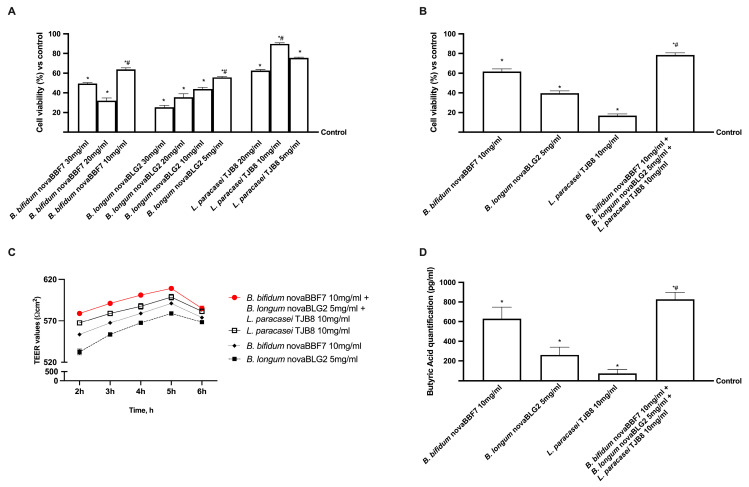
Intestinal effects on Caco-2 cells of probiotics selected. In (**A**,**B**) analysis of cell viability measured by the 3-(4,5-Dimethylthiazol-2-yl)-2,5-diphenyltetrazolium bromide (MTT) test on CaCo-2 cells; In (**C**) TEER values using EVOM3; In (**D**) butyric acid quantification assessed by ELISA kit. Data are expressed as mean ± SD (%) of 5 independent experiments normalised to control. * *p* < 0.05 vs. control; # *p* < 0.05 vs. different concentrations.

Before investigating the possible efficacy of the selected probiotics in influencing cognitive processes, different dosages of *B. bifidum* novaBBF7, *B. longum* novaBLG2 and *L. paracasei* TJB8 strains were assessed in the human intestinal cell model. The mitochondrial metabolism of CaCo-2 cells was analysed in a 24 h dose-response study to screen the best concentration to be used in a new combination. As shown in Figure 1, *B. bifidum* novaBBF7, *B. longum* novaBLG2 and *L. paracasei* TJB8 were able to induce a positive effect on the mitochondrial metabolism compared to the control (*p* < 0.05); in particular, *B. bifidum* novaBBF7 10 mg/mL, *B. longum* novaBLG2 5 mg/mL and *L. paracasei* TJB8 10 mg/mL exerted the greatest effects on cell viability (*p* < 0.05) suggesting that these concentrations could be used for all successive experiments. Precisely, 5 mg/mL of *B. longum* novaBLG2 corresponds to 0.5 × 10^9^ Colony-forming units (CFU)/mL of probiotics, 10 mg/mL of *L. paracasei* TJB8 corresponds to 3 × 10^9^ CFU/mL of probiotics, and 10 mg/mL of *B. bifidum* novaBBF7 corresponds to 1 × 10^9^ CFU/mL probiotics.

Before going into detail about the gut-brain axis, further investigations were performed to gain more information on physiological absorption by performing a gut barrier model validated by the European Medicines Agency (EMA) and Food and Drug Administration (FDA). Therefore, cell viability, transepithelial resistance (TEER) value and metabolite production were evaluated to demonstrate the ability of the new formulation hypothesised to maintain the correct intestinal physiology without inducing cell damage. As shown in Figure 1, cell viability increases for all three concentrations tested compared to the control. However, the greatest effect (*p* < 0.05) was observed with *B. bifidum* novaBBF7 10 mg/mL, *B. longum* novaBLG2 5 mg/mL and *L. paracasei* TJB8 10 mg/mL (approximately 33% vs. *B. bifidum* novaBBF7; 1 time more vs. *B. longum* novaBLG2 and 2 times more vs. *L. paracasei* TJB8), suggesting that the combination is safe for the intestinal epithelium. This effect was confirmed by TEER value analysis, which reached a value of approximately 510 ± 10 Ω × cm^2^ for intestinal cells, as reported in the literature [24], demonstrating that the cells formed an intact monolayer after treatments, maintaining correct intestinal homeostasis. In addition, all the probiotics tested produced a metabolite that crossed the intestinal barrier, reaching the plasma environment (*p* < 0.05), as observed by Butyric acid analysis at the basolateral level. In particular, the metabolite production from *B. bifidum* novaBBF7 10 mg/mL, *B. longum* novaBLG2 5 mg/mL and *L. paracasei* TJB8 10 mg/mL appear to follow a similar cell viability trend, showing an increase in short-chain fatty acid (SCFA) production (approximately 33% vs. *B. bifidum* novaBBF7; 3 times more vs. *B. longum* novaBLG2 and seven times more vs. *L. paracasei* TJB8, *p* < 0.05), amplifying the effects exerted by the single agents, supporting the hypothesis of synergistic activity between *B. bifidum* novaBBF7 10 mg/mL, *B. longum* novaBLG2 5 mg/mL and *L. paracasei* TJB8 10 mg/mL.

### 2.2. The Effects of B. bifidum novaBBF7, B. longum novaBLG2 and and L. paracasei TJB8 on the Gut-Brain Axis

**Figure 2:**
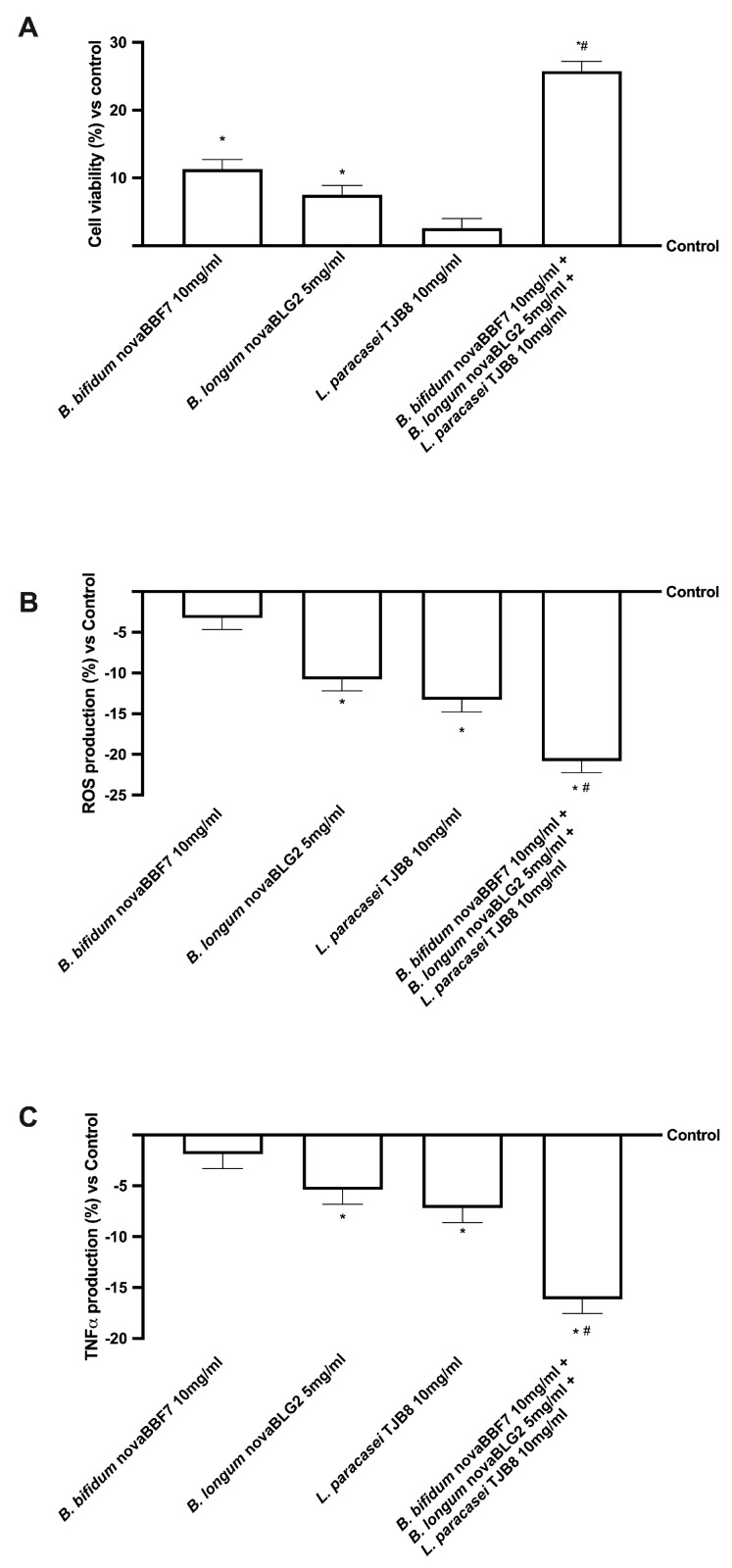
The effects of probiotics on the gut-brain axis. Cell viability was measured through the MTT test (**A**); ROS production was measured by cytochrome C reduction (**B**); TNFα production was measured by an ELISA kit (**C**) on SH-SY5Y cells. Data are expressed as the mean ± SD (%) of 5 independent experiments normalised to control. * *p* < 0.05 vs. control; # *p* < 0.05 vs. single probiotic.

Since the hypothesized target site of the probiotic treatments is the brain, further analyses were conducted on neuronal cells by constructing a brain-gut axis model. As shown in Figure 2, all the probiotic strains tested can affect the final target, probably due to the metabolised product, without any negative effect on mitochondrial metabolism or oxidative stress (*p* < 0.05). In particular, the combination of *B. bifidum* novaBBF7 10 mg/mL, *B. longum* novaBLG2 5 mg/mL and *L. paracasei* TJB8 10 mg/mL was able to amplify the cell viability (*p* < 0.05, Figure 2A) compared to the single agents, exhibiting the ability to lower the quantity of the production of reactive oxygen species (ROS, Figure 2B) while simultaneously demonstrating the ability to sustain mitochondrial health. These positive effects were further confirmed by the analysis of tumour necrosis factor α (TNFα), an inflammatory cytokine produced during acute inflammation and responsible for diverse signalling events within cells, leading to necrosis or apoptosis production (Figure 2C). Noteworthy, all the probiotic strains reduce the production of TNFα compared to the control, demonstrating their beneficial effects (*p* < 0.05, except *B. bifidum* novaBBF7 10 mg/mL). Furthermore, the presence of *B. bifidum* novaBBF7 10 mg/mL and *B. longum* novaBLG2 5 mg/mL and *L. paracasei* TJB8 10 mg/mL significantly improved this effect (*p* < 0.05) compared to the single agents, confirming the synergistic effect of the probiotics.

### 2.3. Analysis of the Mechanisms Underlying Cognitive Functions under Oxidative Stress

**Figure 3:**
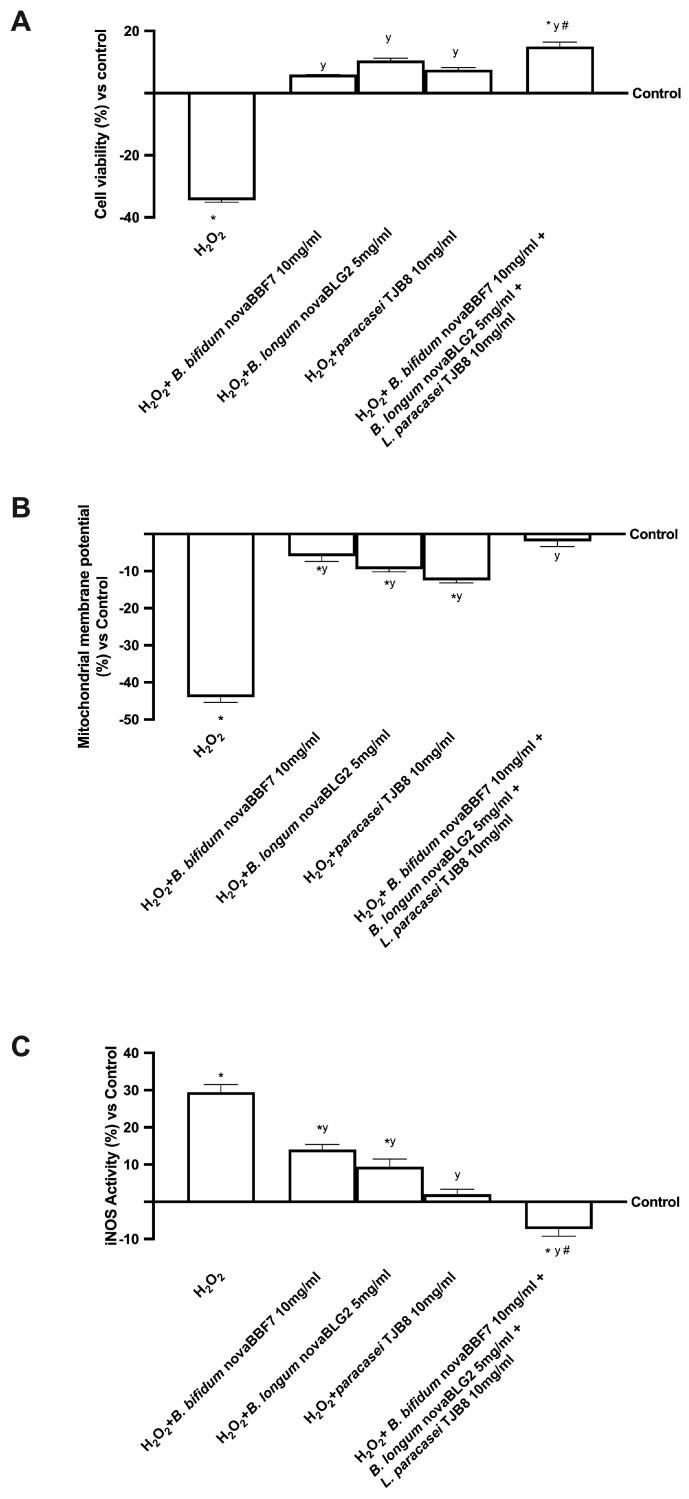
Analysis of the mechanisms underlying cognitive functions under oxidative stress. Cell viability measured through MTT test (**A**); mitochondrial membrane potential (**B**); iNOS activity by ELISA kit (**C**) analysis. Data are expressed as mean ± SD (%) of 5 independent experiments normalised to control. * *p* < 0.05 vs. control; y *p* < 0.05 vs. H_2_O_2_; the bar # *p* < 0.05 vs. other agents.

The potential action of probiotics to prevent cellular damage under oxidative conditions was analysed by cell viability, mitochondrial potential, and inducible nitric oxide synthase (iNOS) in pretreated neuronal cells with 200 μM H_2_O_2_ present at the basolateral level of the gut-brain axis [25]. As shown in Figure 3, exposure to H_2_O_2_ significantly reduced cell viability by approximately 36% compared to the control (*p* < 0.05); contrary, following treatment with probiotic metabolites produced at the intestinal level, the cell viability was significantly increased, but the greatest effect was obtained with *B. bifidum* novaBBF7 10 mg/mL, *B. longum* novaBLG2 5 mg/mL and *L. paracasei* TJB8 10 mg/mL, which reverted the cell loss compared to probiotics alone and compared to H_2_O_2_ (approximately 65%, *p* < 0.05). Moreover, the alteration of the formation of a proton gradient across the inner mitochondrial membrane is considered one of the key indicators of cellular viability. The mitochondrial potential was analysed and, as expected, treatments with all probiotics metabolised, alone and combined, induced a significant increase in JC-1 fluorescence, supporting the active role of probiotics and their combination on mitochondrial activity (*p* < 0.05) also during oxidative stress induced by 200 µM H_2_O_2_. Specifically, H_2_O_2_-treated cells exhibited changes in the fluorescence signal, leading to a decreased red fluorescence signal and an increased green fluorescence signal, indicating a significant dissipation of mitochondrial potential and cell loss compared to the control (*p* < 0.05). Conversely, *B. bifidum* novaBBF7 10 mg/mL, *B. longum* novaBLG2 5 mg/mL and *L. paracasei* TJB8 10 mg/mL in combination reversed the dissipation of mitochondrial potential compared to 200 μM H_2_O_2_ alone (about 3.5 times more, *p* < 0.05), changing the fluorescence signal from green to red. These results indicate that *B. bifidum* novaBBF7 10 mg/mL, *B. longum* novaBLG2 5 mg/mL and *L. paracasei* TJB8 10 mg/mL used in combination produced metabolites able to attenuate the H_2_O_2_-induced apoptosis through the mitochondrial-mediated pathway.

At the same time, since the main theory at the basis of brain degeneration involves the oxidative condition, iNOS expression was investigated. As expected, iNOS expression significantly increased in the presence of 200 μM H_2_O_2_ compared to the control (*p* < 0.05), supporting the hypothesis of the involvement of oxidative stress in neuronal death. On the contrary, the treatment with *B. bifidum* novaBBF7 10 mg/mL, *B. longum* novaBLG2 5 mg/mL and *L. paracasei* TJB8 10 mg/mL alone significantly reduced the expression of iNOS compared to 200 μM H_2_O_2_ alone (*p* < 0.05), but the greater reduction was obtained by their use in combination (approximately 2.5 times more compared to 200 μM H_2_O_2_, *p* < 0.05), indicating a beneficial effect in counteracting the cognitive dysfunctions.

These results indicate that the combination of probiotics can ameliorate cell survival through the gut-brain axis mechanism.

**Figure 4:**
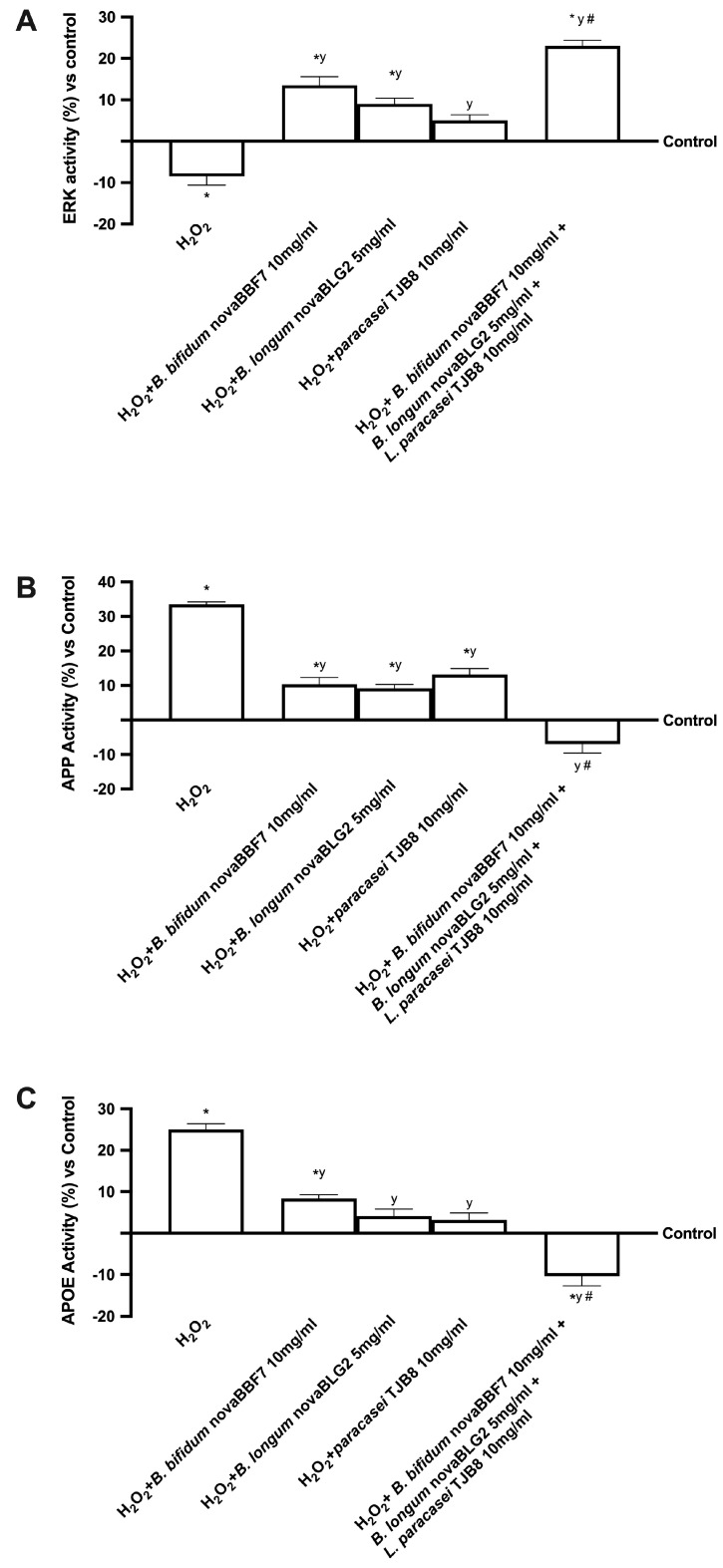
Analysis of the main mechanisms underlying cognitive functions under oxidative stress. ERK (**A**), APP (**B**) and APOE (**C**) analysis by ELISA kit. Data are expressed as mean ± SD (%) of 5 independent experiments normalized to control. * *p* < 0.05 vs. control; y *p* < 0.05 vs. H_2_O_2_; the bar # *p* < 0.05 vs. other agents.

Since the ERK/MAPK pathway plays a crucial role in regulating neuronal and brain survival, additional experiments on its activity were carried out. The treatment of the gut-brain axis with all probiotics alone confirmed their ability to improve viability by activating ERK mediators, as reported in Figure 4. Moreover, the combination of *B. bifidum* novaBBF7 10 mg/mL, *B. longum* novaBLG2 5 mg/mL and *L. paracasei* TJB8 10 mg/mL amplified kinase activation compared to the control and to single administration during exposure to 200 μM H_2_O_2_ (about 3 times more, *p* < 0.05) a more significant effect in ERK/MAPK.

Finally, since a natural consequence of apoptosis is known to be cell loss, the apolipoprotein E (APOE) and β-amyloid analysis (APP) were analysed in the gut-brain axis model. Indeed, H_2_O_2_ caused a significant increase in the APOE and APP activities, supporting previous data about cell death and suggesting impairment in the mechanisms underlying cognitive functions. However, the treatment with probiotics reduced the damage by decreasing APOE and APP activities compared to the control and H_2_O_2_ (*p* < 0.05). Therefore, the most significant effect was obtained when neuronal cells were treated with *B. bifidum* novaBBF7 10 mg/mL, *B. longum* novaBLG2 5 mg/mL and *L. paracasei* TJB8 10 mg/mL (3.5 times more, respectively, compared to 200 μM H_2_O_2_, *p* < 0.05), indicating the effectiveness of the combination during cognitive impairment.

These results demonstrated that *B. bifidum* novaBBF7 10 mg/mL, *B. longum* novaBLG2 5 mg/mL and *L. paracasei* TJB8 10 mg/mL could reverse the damages induced under oxidative conditions, confirming the active role of the gut-brain axis, which can modulate cell loss and cognitive dysfunction.

## 3. Discussion

The relationship between the intestinal tract and CNS is well documented and crucial for the beneficial effects of the gut-brain axis. Indeed, in the current study, we reproduced this axis in vitro to investigate the role of probiotics, after oral intake, in modulating cellular wellness and the main mechanisms involved in cognitive decline. The results demonstrate for the first time that the combination of three probiotics by modulating the exchange of intestinal flow, manages to counteract the neuronal degeneration due to oxidative stress limiting the loss of neuronal cells by acting on specific intracellular mechanisms. In particular, it is important that a combination of probiotics was used to evaluate intestinal homeostasis and its influence on cognitive activities by the gut-brain axis. The results in Figure 1 have shown that the combination of *B. bifidum* novaBBF7 10 mg/mL, *B. longum* novaBLG2 5 mg/mL and *L. paracasei* TJB8 10 mg/mL can successfully influence the intestinal barrier and increase butyrate production than the single agents. In this context, gut microbiota produces numerous metabolites like SCFAs that directly or indirectly affect brain functions. Starting with this first result is important to define the role of the gut-brain axis on cognitive function. Several studies in animal models have proven that the altered gut microbiota is correlated with changes in various neurotrophins and monoamine neurotransmitters, which are key regulators of brain development and plasticity [26]. In addition, ageing-related gut dysbiosis and neurological deterioration are connected because the former is the common cause of a wide range of age-related illnesses by mediating persistent low-grade inflammation [27]. In this context, the gut-brain axis is a network that links the brain’s emotional and cognitive centres to the gut’s regulation and integration of activities and has been linked to the etiology of several psychiatric diseases [27]. To date, increasing research suggests that modifications to the gut microbiota’s composition are a primary cause of several neurocognitive disorders, significantly impacting both CNS immunity and blood-brain barrier (BBB) integrity [28]. Recent evidence has shown that gut microbiota composition is modulated substantially by probiotics supplementation, which has attracted attention in the context of brain function and health because they alter gut microflora toward a beneficial state, which could, in turn, affect the gut-brain axis [29]. Consequently, a growing body of evidence supports the idea that certain probiotics may positively impact the pathogenesis of neuronal disorders. Therefore, the present study established an in vitro model to examine the effect of different probiotics supplementation on the mechanisms underlying cognitive functions. The term cognitive function explored by this study is well described by the concept of the minimal cognitive function [30,31,32,33], which can be used to explore biochemical circuits and network fundaments for biological cognition in neuronal cells.

In this context, it is possible to maintain cognitive function by restoring proper intestinal metabolism, as described by the results obtained from the gut-brain axis. In particular, gut microbiota produces numerous metabolites like SCFAs that directly or indirectly affect brain functions. Butyrate is known to beneficially modulate the peripheral nervous system (PNS) and CNS by inhibiting histone deacetylases and regulating the expression of several genes and proteins [34]. Indeed, an increase in butyrate has been shown to significantly improve learning and memory by amplifying the expression of learning-associated genes in AD mouse models and restoring histone acetylation [35]. Based on the results obtained, it was possible to assume that *B. bifidum* novaBBF7 10 mg/mL, *B. longum* novaBLG2 5 mg/mL and *L. paracasei* TJB8 10 mg/mL, alone and in combination, can directly affect the intended organ by the specific metabolize. Consequently, further experiments were performed considering how this study focused on the gut-brain axis, a bidirectional communication system between the CNS and the enteric nervous system, linking emotional and cognitive centres of the brain with peripheral intestinal functions. Therefore, samples metabolized by intestinal cells were used to stimulate the SHSY-5Y cells placed in the basolateral compartment [36] analyzing the main biological activity exerted by probiotics during cognitive dysfunctions. In detail, the probiotics tested did not induce any damage at the neuronal level, confirming their safety properties and enhancing antioxidant mechanisms and antineuroinflammation activity as revealed by the analysis of cell viability, ROS and TNFα productions in Figure 2, respectively. Specifically, *B. bifidum* novaBBF7 10 mg/mL, *B. longum* novaBLG2 5 mg/mL and *L. paracasei* TJB8 10 mg/mL induced the greatest effects, supporting the hypothesis of synergistic activity between the single agents. In addition, further experiments were performed to evaluate a biological aspect involved in brain ageing and neurodegeneration, such as oxidative stress-dependent damage. Indeed, the role of oxidative stress was investigated by pretreating neuronal cells with 200 μM H_2_O_2_ [37], evaluating the ability of all probiotics, alone and combined, to prevent or restore the damage caused by oxidative stress analyzing cell viability, mitochondrial membrane potential and iNOS activity reported in Figure 3. The results indicate that *B. bifidum* novaBBF7 10 mg/mL, *B. longum* novaBLG2 5 mg/mL and *L. paracasei* TJB8 10 mg/mL, alone and combined, can revert the H_2_O_2_-induced cell loss activating survival pathways. Unsurprising, *B. bifidum* novaBBF7 10 mg/mL, *B. longum* novaBLG2 5 mg/mL and *L. paracasei* TJB8 10 mg/mL once again demonstrated the synergistic effects of the single agents having the greatest benefits on the neuronal cells. Further research was carried out on the neuronal and brain survival pathway’s activity, illustrated in Figure 4. A crucial component of the neuroinflammatory system triggered by glial cells during the onset of neurodegenerative disorders is the MAPK/ERK pathway [38]. Additionally, in this case, the combination of *B. bifidum* novaBBF7 10 mg/mL, *B. longum* novaBLG2 5 mg/mL and *L. paracasei* TJB8 10 mg/mL generated a better effect supporting the hypothesis that all neuronal survival signaling was activated. Specifically, it was able to revert the damages induced by the pretreatment with H_2_O_2_. Finally, since it is known that cell death is a common result of apoptosis, the APOE and APP activities were examined; *B. bifidum* novaBBF7 10 mg/mL, *B. longum* novaBLG2 5 mg/mL and *L. paracasei* TJB8 10 mg/mL was able to revert the damages induced under oxidative condition, demonstrating the probiotics’ active role in treating intestinal dysregulation, which can affect cell death and cognitive impairment. In this setting, the combination of *B. bifidum* novaBBF7 10 mg/mL, *B. longum* novaBLG2 5 mg/mL and *L. paracasei* TJB8 10 mg/mL demonstrated a considerable ability to modulate key cognitive dysfunction pathways with a noticeable synergetic action. Indeed, the stimulation with a combination of three probiotics reflects the ability to modulate the pathways involved in cognitive dysfunction reducing the oxidative stress-related markers, improving survival pathways, and preventing neurodegenerative processes. These effects are more significant with the combination than with the single probiotic alone and are probably linked to the increased butyric acid. For this reason, we can hypothesize that a combination exerts a synergic effect between the single probiotic component derived from different species to support the amplifier effect observed by the combination. The chosen probiotics demonstrate their actual potential application in influencing cognitive processes.

## 4. Materials and Methods

### 4.1. Cell Cultures

The human epithelial intestinal CaCo-2 cell line, purchased from the ATCC (Manassas, VA, USA), was used as an experimental model to predict the features of intestinal absorption following oral intake [39]. This cell line was cultured in Advanced Dulbecco’s Modified Eagle’s Medium/Nutrient F-12 Ham (Adv DMEM-F12; GIBCO^®^ ThermoFisher Scientific, Waltham, MA, USA) containing 10% fetal bovine serum (FBS, Merck Life Science, Rome, Italy), 2 mM L-glutamine, and 1% penicillin-streptomycin (Merck Life Science, Rome, Italy) and maintained in an incubator at 37 °C and 5% CO_2_ [40]. Experiments used cells at passage numbers between 26 and 32 to maintain the correct paracellular permeability and transport properties [41]. The cells were plated differently to perform several experiments, including 1 × 10^4^ cells in 96-well plates to study cell viability using an MTT-based In Vitro Toxicology Assay Kit (Merck Life Science, Rome, Italy). Eight hours before the stimulation, the cells were incubated with Adv DMEM without red phenol and supplemented with 0.5% FBS (GIBCO^®^ ThermoFisher Scientific, Waltham, MA, USA), 2 mM L-glutamine, and 1% penicillin-streptomycin (both from Merck Life Science, Rome, Italy) at 37 °C to synchronize them. In addition, 2 × 10^4^ cells were plated on a 6.5 mm Transwell^®^ (Corning^®^ Costar^®^, Merck Life Science, Rome, Italy) with a 0.4 μm pore polycarbonate membrane insert (Corning^®^ Costar^®^, Merck Life Science, Rome, Italy) in a 24 well plate to perform the absorption analyses [42]. Cells plated on the Transwell^®^ insert were maintained in a complete medium, which was changed every other day on the basolateral and apical sides for 21 days before the simulations [43]. Before the stimulation, on the apical side, the medium was brought to pH 6.5 as the pH in the lumen of the small intestine, while the pH 7.4 on the basolateral side represented blood [44]. This in vitro model is widely used [42] and accepted by the EMA and FDA to predict the absorption, metabolism, and bioavailability of several substances after oral intake in humans [45,46].

SH-SY5Y cells, purchased from the American Type Culture Collection (ATCC, Manassas, VA, USA), were cultured in a mixture of Advanced Dulbecco’s Modified Eagle Medium F12 (Adv DMEM F12; GIBCO^®^ ThermoFisher Scientific, Waltham, MA, USA) and Advanced Dulbecco’s Modified Eagle Medium (Adv DMEM; GIBCO^®^ ThermoFisher Scientific, Waltham, MA, USA) at a ratio of 1:1, supplemented with 10% fetal bovine serum (FBS, Merck Life Science, Rome, Italy), and 2 mM HEPES (Merck Life Science, Rome, Italy), 2 mM L-Glutamine (Merck Life Science, Rome, Italy) and 1% penicillin/streptomycin (Merck Life Science, Rome, Italy). Cells were maintained in a 37 °C incubator at 5% CO_2_ and 95% humidity [47]. The experiments used cells with passage numbers between 3 and 20. The cells were plated differently to perform several experiments, including 1 × 10^4^ cells in 96 well plates to study cell viability by an MTT-based In Vitro Toxicology Assay Kit (Merck Life Science, Rome, Italy), ROS production using cytochrome C (Merck Life Science, Rome, Italy) in a complete medium, TNFα production using an ELISA kit and mithocondrial membrane potential using the JC-1 probe. Eight hours before the stimulation, the cells were incubated with Adv DMEM (GIBCO^®^ ThermoFisher Scientific, Waltham, MA, USA) without red phenol and supplemented with 0.5% FBS (Merck Life Science, Rome, Italy), 2 mM L-glutamine, and 1% penicillin–streptomycin (both from Merck Life Science, Rome, Italy) at 37 °C to synchronize them. In addition, the cells were plated at 4 × 10^5^ cells in 6-well plates to study the intracellular pathways involved, including iNOS activity, ERK, APP, and APOE, using an ELISA kit.

### 4.2. Agents Preparation

*B. longum* novaBLG2 (DSM 34339), *B. bifidum* novaBBF7 (DSM 34336) and *L. paracasei* TJB8 (DSM 33129) donated by Probionova (Lugano, Switzerland) were prepared at the moment. Before performing each stimulation, a different pack of the product was reconstituted by mixing probiotics with DMEM without red phenol (Merck Life Science, Rome, Italy), supplemented with 0% FBS, 50 IU/mL penicillin–streptomycin (Merck Life Science, Rome, Italy) and 2 mM L-glutamine solution (Merck Life Science, Rome, Italy). For each test, performed in triplicate, the samples were diluted in culture medium before being used to reach a final concentration of 0.5 × 10^9^ CFU/mL probiotics, which correspond to 5 mg/mL for *B. longum* novaBLG2, 3 × 10^9^ CFU/mL probiotics, which correspond to 10 mg/mL for *L. paracasei* TJB8, and 1 × 10^9^ CFU/mL probiotics, which correspond to 10 mg/mL for *B. bifidum* novaBBF7.

### 4.3. Experimental Protocol

The experiments were divided into two groups: in the first one, the ability of probiotics to modulate intestinal barrier functions excluding negative effects was analyzed, and in the second one, the effects of probiotic metabolites on intestinal cells were analyzed by the gut-grain axis model to evaluate the intracellular mechanisms underlying the cognitive function in neuronal cells. In the first one, CaCo-2 cell line was used to exclude the cytotoxicity effects of *B. longum* novaBLG2, *B. bifidum* novaBBF7 and *L. paracasei* TJB8, alone and combined, by analyzing mitochondrial metabolism using the MTT test [48]. Subsequently, the best concentration of each probiotic strain was tested on a 3D intestinal in vitro barrier model to verify cell viability using the MTT test and the intestinal stability by TEER analysis, confirming the correct maintenance of the epithelial integrity. Finally, the measurement of butyric acid by ELISA assay was performed to verify the role of one SCFA in cell signaling regulation throughout the entire organism. The cells were treated time-dependent in all these experiments, from 2 h to 6 h [40]. In addition, a gut-brain axis model was created to study the effects of the probiotic strains on physiological conditions. Specifically, the basolateral medium of the intestinal barrier was used to stimulate the neuronal cells for 24 h, the time needed to mimic the correct treatment dosage. At the end of the stimulation, the ROS production and the activation of TNF-α were analyzed. In addition, the effects of probiotic strains were analyzed on a model of neurodegeneration induced through the pretreatment with H_2_O_2_ (Merck Life Science, Rome, Italy)_,_ as reported in the literature [49]. In particular, cell viability, mitochondrial membrane potential and crucial cognitive function pathways such as ERK/MAPK, APOE and APP activity were analyzed under oxidative stress conditions under H_2_O_2_ pretreatment.

### 4.4. Gut-Brain Axis Model

The Transwell^®^ co-culture method with CaCo-2 and SHSY-5Y cell lines was carried out in accordance with a standard protocol described in the literature [50]. A semipermeable membrane with a pore size of 0.4 μm (Corning^®^ Costar^®^, Merck Life Science, Rome, Italy) was used to separate the two chambers filled with DMEM medium (Merck Life Sciences, Rome, Italy). In summary, our insert co-culture model is built as follows: CaCo-2 cells were plated in dense layers on filter inserts (25,000 cells for insert). Lower-density SH-SY5Y cells were plated in an independent 24-well (400 SH-SY5Y cells/well), flat-bottom plate on the seventh development day. In cases where cells were plated at *n* = 400 cells/well and left untreated for 5 days, these neuroblastoma-sized neurites were observed. Instead, stellate forms occur within 24 h of growth when plating *n* = 25,000 cells/well. This occurs because SH-SY5Y cells promote the growth of one another.

At 14 days following intestinal epithelium maturation, the cells on the culture media will have acquired a high TEER value, which is suggestive of tight junction development (≥500 Ω·cm^2^). Both cell lines were grown separately for an additional 5 days. Then, the two cell lines were placed together for 15 h in the incubator. To prevent potential modification of the intestinal cell monolayer, TEER was measured once more when the two lines were combined before stimulation. After that, cell viability tests, quantification of ROS production, and evaluation of mitochondrial metabolism during the brain degenerative process were performed on the cells.

### 4.5. MTT Test

At the end of stimulation, the MTT test was performed as described in the literature [51] to determine cell viability. Cells were incubated in DMEM without phenol red, 0% FBS with 1% MTT dye for 2 h at 37 °C in an incubator, 5% CO_2_ and 95% humidity, and then cell viability was determined by measuring the absorbance through a spectrometer (Infinite 200 Pro MPlex, Tecan, Männedorf, Switzerland) at 570 nm with correction at 690 nm. The results were obtained by comparing them with control cells (100% viable).

### 4.6. Intestinal Integrity Analysis

The TEER values of the Caco-2 cells plated on the inserts were continuously measured on alternate days for 21 days using EVOM3 (World Precision Instruments, Sarasota, FL, USA), and the experiments were started when TEER reached ≥500 Ω·cm^2^. In the literature, it is reported that TEER values ≥ 500 ± 52.9 Ω·cm^2^ are recommended for the transport study [40].

### 4.7. Butyric Acid Quantification

The butyric acid produced after stimulation of Caco-2 cells with probiotics was quantified with an ELISA kit (Cloud-Clone, Wuhan, China) according to manufacturer instructions [52]. The absorbance of each sample was measured after the addition of stop solution at 450 nm using a plate reader (Infinite 200 Pro MPlex, Tecan, Männedorf, Switzerland). The OD was interpolarized with a standard curve (from 10.000 pg/mL to pg/mL), expressing the data as mean (pg/mL) compared to control.

### 4.8. ROS Production

The quantification of superoxide anion release was obtained following a standard protocol based on the reduction in cytochrome C [51], and the absorbance in culture supernatants was measured at 550 nm using the spectrophotometer (Infinite 200 Pro MPlex, Tecan, Männedorf, Switzerland). Specifically, 100 μL of cytochrome C (Merck Life Science, Rome, Italy) was added to all the wells, while 100 μL of superoxide dismutase (Merck Life Science, Rome, Italy) and 100 μL of cytochrome C were added to empty wells and the plate was then incubated for 30 min. After that, 100 μL was taken from each well and the absorbance was measured with a spectrophotometer (Infinite 200 Pro MPlex, Tecan, Männedorf, Switzerland) at 550 nm. The O_2_ rate was expressed as the mean ± SD (%) of nanomoles per reduced cytochrome C per microgram of protein compared to the control (0 line).

### 4.9. TNFα Assay Kit

TNFα production on SHSY-5Y cells under oxidative stress was analyzed by the Human Tumor Necrosis Factor α ELISA Kit (Merck Life Science, Rome, Italy) following the manufacturer’s instructions. Briefly, 100 µL of SHSY-5Y’s lysate was added to each well of a 96-well ELISA plate, and the plate was incubated at room temperature for 2 h, followed by overnight incubation at 4 °C. At the end of incubation, wells were washed five times with a washing buffer, and 100 μL of biotinylated anti-TNFα was added to each well. After 2 h of incubation at room temperature, the solution in each well was aspirated, the wells were washed five times and 100 μL of streptavidin-HRP was added to each well and incubated at room temperature for 1 h. After washing, 100 μL of chromogen solution was added to each well and incubated for 30 min at room temperature and in the dark. The absorbance of each well was measured after the addition of stop solution at 450 nm using a plate reader (Infinite 200 Pro MPlex, Tecan, Männedorf, Switzerland) [50].

### 4.10. Mitochondrial Membrane Potential

The Oxygen Consumption/Mito membrane Potential Dual Assay Kit (Cayman Chemical Company, Ann Arbor, MI, USA) analyzed the mitochondrial membrane potential by following the manufacturer’s instructions [37]. The mitochondrial membrane potential was measured using JC-1 aggregates at an excitation/emission of 560/590 nm and monomers at an excitation/emission of 485/535 nm in a fluorescence spectrometer (Infinite 200 Pro MPlex, Tecan, Männedorf, Switzerland). The results are expressed as (%) compared to control cells of SHSY-5Y.

### 4.11. iNOS ELISA Kit

iNOS activity was determined using an ELISA kit (Thermoscientific, Waltham, MA, USA) to verify the iNOS presence in cell lysates of SHSY-5Y, according to the manufacturer’s instructions [53]. The samples were analyzed by a spectrometer (Infinite 200 Pro MPlex, Tecan, Männedorf, Switzerland) at 450 nm. The concentration is expressed as ng/mL compared to a standard curve (range from 0.4 to 100 ng/mL), and the results are expressed as percentage (%) versus control (0 line).

### 4.12. ERK/MAPKS ELISA Kit

The ERK/MAPK activation was measured by the InstantOneTM ELISA (Thermo Fisher, Milan, Italy) on SHSY-5Y lysates, as reported in the literature [37]. At the end of treatment, the cells were lysed with 100 μL Cell Lysis Buffer Mix, shaken for 10 min at room temperature and then 50 μL/well of each sample was tested in InstantOne ELISA microplate strips. At each well, 50 μL of prepared antibody cocktail was added, and the strips were incubated for 1 h at room temperature on a microplate shaker and washed 3 times with 200 μL/well of Wash Buffer. At the end, 100 μL of the Detection Reagent was added to each well, and after 20 min the reaction was stopped by adding 100 μL of Stop Solution. The strips were measured by a spectrometer at 450 nm (Infinite 200 Pro MPlex, Tecan, Männedorf, Switzerland). The results were expressed as mean absorbance (%) compared to control.

### 4.13. APOE 9 ELISA Kit

According to the manufacturer’s instructions, APOE activity was determined using an ELISA kit (Thermoscientific, Waltham, MA, USA) in cell lysates of SHSY-5Y, according to the manufacturer’s instructions. 100 µL of standards were added to the appropriate wells to create a standard curve. For samples, we added 100 µL of diluted samples to the wells, and then the wells were covered and incubated for 2.5 h at room temperature or overnight at 4 °C with gentle shaking. At the end of incubation, 100 µL of prepared biotin conjugate were added to each well. The samples were incubated for 1 h at room temperature with gentle shaking. Then, 100 µL of prepared Streptavidin-HRP solution was added to each well and incubated for 45 min at room temperature with gentle shaking. Then the solution was discarded, and the samples were washed. 100 µL of TMB Substrate was added to each well. The substrate will begin to turn blue. Samples were incubated for 30 min at room temperature in the dark with gentle shaking. In the end, 50 µL of stop solution was added to each well, and the solution was mixed and changed from blue to yellow [53]. The samples were analyzed by a spectrometer (Infinite 200 Pro MPlex, Tecan, Männedorf, Switzerland) at 450 nm. The concentration is expressed as ng/mL compared to a standard curve (range from 1.6 to 400 ng/mL), and the results are expressed as percentage (%) versus control (0 line).

### 4.14. APP ELISA Kit

APP quantification was measured by the Amyloid Beta A4 protein ELISA kit (Merck Life Science, Rome, Italy) on cellular supernatants of SHSY-5Y cells, as reported in the literature [37]. Briefly, at the end of treatments, cellular supernatants were collected, and each sample was tested with the ELISA kit. The biotinylated detection antibody specific to the target protein was added to each well, and the plate was incubated for 1 h at room temperature. Then, after 45 min of incubation with HRP-conjugated streptavidin, TMB substrate solution was added for 30 min, and subsequently, the reaction was stopped by adding a stop solution. APP concentration was determined by measuring the absorbance through a spectrometer (Infinite 200 Pro MPlex, Tecan, Männedorf, Switzerland) at 450 nm. The concentration was calculated by comparing the results to the APP standard curve.

### 4.15. Statistical Analysis

Data collected were processed using Prism GraphPad statistical software 9.4.1 (GraphPad Software, La Jolla, CA, USA) using one-way analysis of variance (ANOVA), followed by Bonferroni post hoc tests. Comparisons between the two groups were performed using a two-tailed Student’s *t*-test. Multiple comparisons among groups were analyzed by a two-way ANOVA followed by a two-sided Dunnett post hoc test. All results were expressed as the mean ± SD of at least 5 independent experiments produced in triplicate. Differences with a *p* < 0.05 were considered statistically significant.

## 5. Conclusions

The study demonstrated that probiotics could modulate the gut-brain axis, restoring the proper intestinal metabolism, which is related to maintaining cognitive functions. *B. bifidum* novaBBF*7*, *B. longum* novaBLG2 and *L. paracasei* TJB8 improve intestinal homeostasis by improving brain activity and decreasing cell loss at the neuronal level. Moreover, SCFAs secreted by probiotics, such as butyric acid, can act as second messengers and activate various mechanisms otherwise impaired in conditions of cognitive dysfunction. Therefore, this study suggests that maintaining a healthy intestinal microbiota through the supplementation of probiotics such as *B. bifidum* novaBBF*7*, *B. longum* novaBLG2 and *L. paracasei* TJB8 aids in maintaining cognitive functions.

## Data Availability

Raw data are preferably deposited at the Laboratory of Physiology (C. Molinari), ensuring appropriate measures so that raw data are retained in full forever under a secure system. The data presented in this study are available upon reasonable request from the corresponding author.

## Abbreviations

AD	Alzheimer’s disease
ADV DMEM	advanced Dulbecco’s Modified Eagle’s Medium
ADV DMEM-F12	advanced Dulbecco’s Modified Eagle’s Medium/Nutrient F-12 Ham
APOE	apolipoprotein E
APP	β-amyloid analysis
BBB	blood-brain barrier
BDNF	brain-derived neurotrophic factor
CFU	colony-forming unit
CNS	central nervous system
EMA	European Medicines Agency
ERK	extracellular signal-regulated kinase
FBS	fetal bovine serum
FDA	Food and Drug Administration
GABA	gamma-aminobutyric acid
HRP	horseradish peroxidase
iNOS	inducible nitric oxide synthase
MAPK	mitogen-activated protein kinase
MCI	mild cognitive impairment
MTT	3-(4,5-Dimethylthiazol-2-yl)-2,5-diphenyltetrazolium bromide
PNS	peripheral nervous system
SCFAs	short chain fatty acid
TEER	transepithelial electrical resistance
TMB	3,3′,5,5′-tetramethylbenzidine
TNFα	tumor necrosis factor α
